# Personal Exposure to Social Media and Variations by Gender among Cuban Youth

**DOI:** 10.11621/pir.2023.0405

**Published:** 2023-12-01

**Authors:** Jorge Enrique Torralbas Oslé, Emely Corcho Rosales

**Affiliations:** a University of Havana, Cuba

**Keywords:** digital socialization, personal exposure, youth, social media, disorders

## Abstract

**Background:**

Personal exposure is a crucial aspect of digital socialization. It pertains to the amount of time spent on social networks, the number of active accounts, interactions on different platforms, the content published on social networks, the purpose for which the content is shared, the degree of personal exposure, and the changes in social life caused by the exposure. Gender plays an important role in predicting online behavior, but previous studies have yielded contradictory results.

**Objective:**

To characterize personal exposure to social media in young Cubans (networks used, hours of exposure, published content, how much of one’s private life is exposed, alterations due to its use). To define the differences in personal exposure based on the gender variable.

**Design:**

A survey specifically created for this research was used. The sample was composed of 3345 young Cuban residents between 18 and 35 years old.

**Results:**

The young people spent a considerable amount of time per day on WhatsApp, Instagram*, and Facebook/Messenger*. Users shared content related to humor, music/art, and their personal lives. These interactions served as a source of entertainment, a means of communication and socialization, and a platform for expressing opinions on various topics. Personal content was shared frequently. The young people reported experiencing disturbances such as family demands, decreased attention span, difficulty sleeping, and decreased social interactions. There were gender differences in the level of personal exposure, with females reporting higher levels of exposure.

**Conclusion:**

There are high levels of personal exposure among Cuban youth. Different qualities are manifested according to gender. Females reflect the highest levels of personal exposure.

## Introduction

The use of digital technologies, particularly social media, is steadily increasing across all age groups, especially among the youth (Chaffey, 2016; Koç et al., 2019). Social media provides young people with a sense of freedom to express themselves, a platform to make new friends and maintain regular contact with them, and an opportunity to create different communities.

Digital socialization refers to the process of socialization that occurs through technological means such as online platforms (Soldatova et al., 2020). It involves the acquisition of social experiences and the transmission of social identity, which includes traditions, culture, and social roles. Personal exposure is an essential dimension of this process, which refers to the sharing of personal life and intimacy on digital platforms, such as photographs, videos, and opinions. The following parameters can be used to analyze digital socialization: hou rs spent on social networks; number of active accounts; interactions on different platforms; content published on social networks; the purpose for which content is shared; the degree of personal exposure; the level of concern about one’s projected image on digital media; and alterations in social life as a result of the exposure.

Digital socialization is a socialization mediated by the available technological processes and constituting the appropriation of social experiences acquired online (Soldatova et al., 2020). It is a phenomenon with both an individual and social character; it enables the transmission of a social identity — *i.e.,* tradition, culture, and social roles — among other things (Balea-Fernández, 2021). Personal exposure is one essential dimension of this process.

Social media, like other spaces of primary socialization, has a significant impact on the process of personality formation and a person’s worldview. However, social media differs from other social spaces in various ways. For instance, it offers immediacy, allowing users to communicate with several people simultaneously while carrying out daily tasks. Social media also provides the ability to view and share private information beyond one’s immediate circle, anonymity when desired, new codes of nonverbal communication, and ways of expressing emotions symbolically. However, it can give users a false sense of control over the situation and lead to the user losing a sense of time (Hodkinson, 2017; Marder et al., 2016; Santos, 2018; Pashkovsky, 2019; Prete & Redon, 2020). Social media is a new form of existence in time and space, with peculiarities concerning the configuration of identity, subjectivity, and forms of relationship.

Various studies which have explored the utilization of digital technologies among young adults, have found that these users are highly and consistently engaged with social media platforms. These results are indicative of significant cultural transformations, which are characterized by the wide-ranging adoption of digital technologies (Abi-Jaoude et al., 2020; Cantor-Silva et al., 2018; Goodyear & Armour, 2019; Golovchin, 2022; León et al., 2022; Prete & Redon, 2020).

Understanding people’s online activities and their consequences is crucial. Online behavior can be directed towards various activities such as acquiring information, reading news, enhancing personal growth, socializing, entertainment, shopping, and playing games with different levels of intensity (Mude & Undale, 2023; Ryan et al., 2014; van Deursen & van Dijk, 2014). Gómez (2020) proposes four techno-social dimensions that mediate this process: motivation, degree of formality, degree of sociality, and type of technological domestication.

Personal data is becoming increasingly relevant and frequently shared, which raises an important issue. There are different consequences, especially for the child and youth population. Exposure to curated content may create an unrealistic view of others’ lives, leading to feelings of inadequacy or low self-esteem. Reduced face-to-face interactions, increased screen time, and sedentary lifestyles may result in issues like eye strain, poor sleep quality, and validation-seeking behavior. Additionally, loss of personal privacy may potentially expose individuals to various risks, which can impact mental health. And these are only some of the potential problems. (Abi-Jaoude et al, 2020; Allahverdi, 2022; Beyens et al., 2016; Boer et al., 2022; Munar, 2010; Onete et al., 2020)

Different people interact online in different ways. Studies on the digital divide have shown that people from different genders, age groups, educational backgrounds, and experiences use the Internet in different ways. Among these factors, gender has been found to be a particularly important predictor of online behavior (van Deursen & van Dijk, 2014).

Studies have shown that men tend to spend more time on social media compared to women. However, the differences are not limited to the amount of time spent on social media, but also to the effects the exposure has on them. Despite women spending less time on social media, it has a greater impact on their academic performance compared to men. (Ali et al., 2021; Alnjadat et al. 2019; Liu, 2018; Schodt, 2021). In contrast, other studies point to greater use by women of Snapchat, Facebook*, and Instagram*, as well as a greater focus on maintaining and building a wider network of contacts, while men move more toward other digital spaces such as video games (Allison et al., 2017; Kasahara et al., 2019; Metastasio et al., 2016).

Research on the topics mentioned above is growing, but still scarce in Cuba, which presents a unique context. Mobile data usage in the country was limited until December 2018, when it was activated for general use in a limited way. However, it only became widespread and more affordable during the COVID-19 pandemic. Over time, there has been a steady increase in Internet penetration of the population, which currently stands at 68%, mostly through cellular telephony. This accelerated process, in a short period, makes Cuba a unique case, but one that can provide significant information. What are the characteristics of social network exposure of young Cubans? Is it similar to or different from that of other contexts?

The aim of this study was to analyze the personal exposure of young Cuban individuals to social media in the period from May to July 2022. The study focused on how gender influenced the understanding of this process and primarily analyzed the levels of personal exposure.

*Hypothesis 1*: It is hypothesized that there are high levels of personal exposure, which show up in the amount of time young people spend on social networks daily, the content they publish about their private lives, and the negative impact on their social life.

*Hypothesis 2*: Personal exposure varies based on gender. There are specific differences in the content males and females tend to publish, their motivations for it, and the time they spend on social networks. These differences are influenced by social and cultural factors, and result in varying levels of personal exposure for males and females.

## Methods

### Participants

A sample of 3,345 young people was assembled in a non-probabilistic manner by quotas. A specific number of participants per province was defined, calculated so as to be representative of the population, as indicated by the data obtained by the Oficina Nacional de Estadística e Información (2022). Based on this analysis, a distribution by cluster was made, so that the sample was calculated with 99% representativeness and a 4% margin of error.

The ages of the subjects in the sample ranged from 18 to 35 years, because that is the period that is understood in Cuba to comprise Youth (M = 22.6, SD = 4.0); 63.3% were females and 36.7% were males.

### Instruments

The research relied on a quantitative methodology, with a comparative and exploratory approach. This methodology was chosen to gather comprehensive and wide-ranging data on the phenomenon across the country. The authors of the research created a survey that was not subject to validation since it was designed to collect global information on the investigated phenomenon, rather than to standardize it.

The survey had 10 questions: one on sociodemographic data; a closed question on time spent on social networks; five multiple-choice questions that explored the users’ networks, content posted, purpose, and alterations; and a Likert-type scale question, which explored concern for personal image (see Appendix).

### Procedure

The data collection process consisted of two phases. The first phase involved a pilot test which was administered to 60 people. Based on their feedback, some questions were modified and others were converted into closed-ended questions, resulting in the final version of the survey. In the second phase, a survey was conducted online between May 23 and July 6, 2022. It was disseminated through various digital channels using the snowball method to reach a wide audience.

A study was conducted using 100 questionnaires to compare the results obtained through face-to-face interviews, digital surveys, and self-administered surveys. The study found that there were no significant differences in the results between the online and self-administered surveys, and the face-to-face interviews.

Data analysis was carried out using the Statistical Package for Social Sciences IBM SPSS V22. To test Hypothesis 1, descriptive statistics such as frequencies and means were used; for Hypothesis 2, nonparametric tests like Chi-square and Mann-Whitney U were employed. Additionally, a simple correspondence analysis was performed to investigate the relationship between social networks, type of content posted, and gender.

## Results

The study found that a vast majority of young people are extensively using digital social media. Specifically, almost all of the participants reported using social media, with more than half of them spending over five hours a day on these platforms. Moreover, a significant number of respondents were found to use social media for more than 10 hours a day. It was observed that females tended to spend more time on social media compared to males: X^2^ (3, N = 3344) = 66.35, *p*<.001 (see *Table 1*).

**Table 1:** Time spent on social media

Time spent	Male		Female		Total	
	Frequency	Percent	Frequency	Percent	Frequency	Percent
Does not use	9	0.7	9	0.4	18	0.5
1hr — 4hrs	628	51.2	814	38.4	1442	43.1
5hrs — 9hrs	477	38.9	950	44.9	1427	42.7
More than 10hrs	112	9.1	345	16.3	457	13.7
Total	1226	100.0	2118	100.0	3344	1000

Socialization through WhatsApp was shared and generalized by almost the entire sample. There was a difference according to gender in the most used networks: X^2^ (9, N = 3344) = 528.87, *p*<.01. By comparison, females had a higher usage of Instagram*, Facebook/Messenger*, Pinterest, and TikTok/Likee, while males were more likely to use Telegram, You Tube, and Twitter* (see *Table 2*).

**Table 2:** Social media on which they were present

Social media	Male		Female		Total	
	Frequency	Percent	Frequency	Percent	Frequency	Percent
WhatsApp	1198	97.7	2106	99.4	3304	98.8
Instagram*	563	45.9	1370	64.7	1933	57.8
Facebook/Messenger*	583	47.6	1296	61.2	1879	56.2
Telegram	621	50.7	704	33.2	1325	39.6
You Tube	433	35.3	454	21.4	887	26.5
Twitter*	330	26.9	322	15.2	652	19.5
Pinterest	112	9.1	424	20.0	536	16.0
TikTok/Likee	26	2.1	122	5.8	148	4.4
LinkedIn	28	2.3	31	1.5	59	1.8
Total	1226	100.0	2118	100.0	3344	100.0

The most common ways these youth were exposed to content online included chatting (70.6%), communicating stories or status updates (60.1%), reacting to posts (33.3%), posting content (24.9%), leaving comments (23.5%), and sharing content (21.2%). These types of interactions are typically active and short-lived, such as conversations or stories that disappear within 24 hours.

Almost all the participants reported publishing content on social media, with a majority of them sharing content related to humor, music/art, and their personal lives. The most recent content published on social media platforms was found to be associated with religion. There were noticeable differences in the content published by males and females: X^2^ (13, N = 3320) = 668.29, p<.01. Males tended to publish more content related to humor, sports, science, and politics and were less inclined to share information about their personal lives. On the other hand, females were more likely to share content related to their personal lives, buying/selling, work/study, motivation, and fashion/trends (see *Table 3*).

**Table 3:** Content they post about on social media

Content	Male		Female		Total	
	Frequency	Percent	Frequency	Percent	Frequency	Percent
Humor	851	70.0	1313	62.4	2164	65.2
Music/Art	413	34.0	686	32.6	1099	33.1
Private life	239	19.7	767	36.5	1006	30.3
Work/Study	297	24.4	700	33.3	997	30.0
Motivational	178	14.6	552	26.2	730	22.0
Buying/Selling	200	16.4	503	23.9	703	21.2
Science	231	19.0	255	12.1	486	14.6
Promotion	139	11.4	304	14.4	443	13.3
Politic	230	18.9	194	9.2	424	12.8
Fashion and trends	93	7.6	301	14.3	394	11.9
Sport	297	24.4	95	4.5	392	11.8
None	91	7.5	121	5.8	212	6.4
Religion	45	3.7	71	3.4	116	3.5
Total	1216	100.0	2104	100.0	3320	100.0

There was an association between the content published and the social media platforms where the youth were most involved: X^2^ (119, N = 3320) = 1196.27, *p*<.01. Facebook*, Instagram*, and Pinterest were more commonly associated with content about the youth’s private life, buying/selling, motivation, fashion, trends, music, and art. This kind of content had a higher female audience. By contrast, YouTube, Telegram, Twitter*, and LinkedIn had more content related to sports, science, and politics, and a higher male audience.

Social media was mainly used by the participants as a source of entertainment (71.4%), a platform to share and distribute content that is of interest to others (49.9%), and a way to communicate and socialize (37.0%). Participants also used social media as a space for expressing their thoughts on various topics (32.5%) and promoting their study or work (29.7%). However, social media was rarely used for buying or selling activities, offering help, or sharing personal life details.

Participants mainly shared topics related to their friendships (49.4%), spare time (49.0%), family (38.4%), study and/or work (35.8%), personal interests (28.3%), love interest (25.7%), and socio-political activities (7.1%).

Although it is not clear why the youth shared their private lives with others, the data suggests that it happens quite frequently. Only 20% of the participants stated that they never share their private content, while almost 50% reported doing so several times a month. There were also significant gender-related differences in this regard. The data shows that females tended to share more private content than males: X^2^ (6, N = 3344) = 122.41, *p*<.01. This indicates that people use social media to satisfy their need for recognition by sharing their private content with others. You can refer to *Table 4* for more information (see *Table 4*).

**Table 4:** Frequency of publication of contents of private life

Frequency of publication about private life	Male		Female		Total	
	Frequency	Percent	Frequency	Percent	Frequency	Percent
Never	370	30.2	328	15.5	698	20.9
Every several months	248	20.2	573	27.1	821	24.6
Once a month	121	9.9	290	13.7	411	12.3
Several times a month	217	17.7	511	24.1	728	21.8
1–3 time a week	172	14.0	275	13.0	447	13.4
Every day	52	4.2	83	3.9	135	4.0
Several times a day	46	3.8	58	2.7	104	3.1
Total	1226	100.0	2118	100.0	3344	100.0

Although the respondents acknowledged that they mostly published about their private lives, the frequency of publication varied significantly. A significant portion of the sample stated that they publish about their private lives more than once a month, ranging from several times a day to several times a month. However, another group of approximately the same number of people stated that they publish every few months or never.

In general, the youth were highly concerned about the image they projected on social networks. Participants paid close attention to the content others post about them and were worried about the image they portray of themselves. Females showed more intense concern than males in these respects. These young people were also concerned about other people’s comments and publications about them, albeit to a lesser extent (see *Table 5*).

**Table 5:** Concern about the image projected on social media

Proposición	Male	Female	Global	Mann-U Whitney de	Sig. (bilateral) asintót.
I don’t like others to post any image, comment or video of mine without consent	3.81	4.03	3.95	1180915.50	.000
I select my best images to post on my profiles	3.55	3.98	3.83	1097595.00	.000
I give importance to what is said about me on social networks	2.69	2.73	2.72	1279414.50	.470

There was a correlation between the frequency of sharing private life content and the level of concern about the image projected: X^2^ (6, N = 3344) = 277.68, p<.01. Participants who showed their private lives less were more concerned about the publication of images without their consent.

The sample of young people in this study acknowledged the various negative effects of social media use. They reported that their families complained about the amount of time they spent online, and they themselves reported experiencing a decreased attention-span and concentration, difficulty sleeping, and less social interaction. Women who used social media more frequently showed higher levels of negative effects compared to men: X^2^ (8, N = 3319) = 91.23, *p*<.01. It is alarming to note that 10% of the participants reported experiencing three or more of these negative effects simultaneously (see *Table 5*).

**Table 6:** Perceived disorders resulting from presence on social media

Disorders	Male		Female		Total	
	Frequency	Percent	Frequency	Percent	Frequency	Percent
Family’s claim time for connection	362	29.9	839	39.8	1201	36.2
None	473	39.1	644	30.6	1117	33.7
Decrease concentration in attention and	350	28.9	673	31.9	1023	30.8
Difficulty sleeping well	259	21.4	513	24.3	772	23.3
Decreased social interactions face-to-face	240	19.8	474	22.5	714	21.5
Feelings and/of anxiety, or distress insecurity	195	16.1	442	21.0	637	19.2
Social isolation	142	11.7	318	15.1	460	13.9
Alterations behaviors in nutritional	72	5.9	166	7.9	238	7.2
Total	1211	100.0	2108	100.0	3319	100.0

It has been found that the amount of time young people spend on social media is significantly related to the development of certain disorders. As the perceived number of hours spent on social networks increases, so does the likelihood of experiencing alterations in behavior. Individuals who realize that they spend more than 10 hours a day on social media are more likely to experience a greater number of disorders. Increased exposure to social media can lead to family complaints, feelings of anxiety and insecurity, and difficulty sleeping: X^2^ (24, N = 3319) = 560.55, p<.01.

## Discussion

The study’s results support the two hypotheses. Hypothesis 1 stated that there are high levels of personal exposure among Cuban youth, which is evident in the amount of time these young people spend on social media and the content they are exposed to, especially private life content. Social media are more than just a tool for work or consumption; they are also a means of fulfilling needs, which makes young people consider them to be a space where they can express themselves and modify the forms of their relationships. Social media not only interact with content and imitate it in real life, but they also reflect people’s values and lifestyles. As suggested by Prete & Redon (2020), the narrative people project online reflects subjective elements of the individual’s reality.

Social media provide entertainment, and an outlet for expression, communication, content interaction, product promotion, and academic/labor activity (Abi-Jaoude et al., 2020; Candale, 2017; Colás-Bravo, et al., 2013; Domínguez & López, 2015). The expression of opinions and ideas is a crucial aspect of social media use, which satisfies the need for subjective meaning-making. Moreover, activity on the Internet contributes significantly to psychological well-being, especially when it involves interactions with others (Goodyear & Armour, 2019; Nikitina, 2021).

Young people have a tendency to showcase their private lives on social media, projecting their ways of thinking and living, which sometimes creates an idealized image of themselves. Our participants acknowledged that they tended to use social media to embellish the truth and present a perfect image of their lives. They used the platform to share their activities with others. Publishing private information not only serves as a means of expression. Rather, the online presence of an individual helps to maintain the integrity of their personality and identity in the social media realm. It also reflects the image that its creator desires to project (Pogorelov & Rylskaya, 2022).

It is important to note that the high levels of concern for one’s image online can be attributed to the significant involvement and exposure of young people to social networks. Additionally, it highlights the awareness of the risks associated with the use of social media, particularly in regard to privacy issues.

Based on the results, it is evident that social media interactions are short-lived and fleeting in nature. The focus is on instant gratification and immediate responses. This type of participation and socialization carries a risk of decreasing users’ levels of reflection and gradually losing track of history (Abi-Jaoude et al., 2020; Soldatova, et al., 2022).

The specific ways in which people are exposed to various environments can lead to either positive or negative outcomes, just as other social situations do. Our research highlighted the challenges involved in adapting to different social environments, which can result in significant changes, particularly at the cognitive and emotional levels.

Based on Hypothesis 2, personal exposure exhibits different characteristics depending on gender. Women tend to reflect higher levels of personal exposure, which leads to a higher degree of alteration, including family claims and decreased concentration levels. While most studies have concluded that the differences between men and women are not related to the amount of use, but to certain characteristics of such use, this study revealed a difference that goes both ways (Espinar & González, 2009; Liu, 2018).

It has been observed that women tend to use social networks more than men. They are more active in terms of publishing and consuming content related to their private life, work/study, fashion and trends, motivation, and buying/selling on platforms like WhatsApp, Instagram*, and Facebook*. They also tend to share their private lives more frequently, which makes them more concerned about the image they project. This aligns with previous studies that indicated that women use social networks more for personal care, employment, and training. Additionally, they prefer social networks that require the publication of images. However, this contradicts other studies that claim that men spend more time consuming content on social media (Valencia et al., 2020; Espinoza & Chávez, 2021).

The differences between men and women in the content they post, their motivations, and the time they spend on social media show that these digital spaces are not separate from the sociocultural context of face-to-face interactions. Gender can influence the process of socialization, leading to differences in the way people interact online. This also means that the stereotype that men are more reserved online and only interested in topics like sports, science, and politics is perpetuated.

This information is very relevant both theoretically and practically. These results are very similar to those from contexts with different social, economic and political organizations. They were obtained after only five years of mobile data use. Also, they occurred in a context of limited digital socialization due to issues of access to certain platforms and still limited connectivity. This speaks to the depth of the impact of digital socialization on social networks and the challenges they present in the globalization of living standards and relationships. Therefore, these results are in themselves relevant.

## Conclusions

There is a trend of overexposure to social media interactions, mostly on WhatsApp, Facebook*, and Instagram*. People engage in discussions about humor, music/art, and their personal lives, such as friendships, free time, and family. This is mainly done using images and memes through ephemeral interactions. The participants are highly concerned about the image they project and tend to share a lot of their personal life, which leads to various disturbances in their daily life, such as family issues and decreased attention span and concentration. Females tend to share more of their personal lives, leading to higher levels of personal exposure.

The exhibition of private and intimate life on the Internet reflects the attempt to be part of cyberspace, and affects the balance between face-to-face and virtual socialization spaces. This translates into alterations in social life, which, in turn, can expose youth to online risks to a greater extent. Therefore, it is necessary for future studies to analyze the phenomenon from a mixed or qualitative methodology that allows researchers to deepen our understanding of the ways in which young people are exposed to social networks, and their affective, cognitive, and behavioral impact, as well as to analyze the possible online risks that may result from such levels of exposure.

## Limitations

The main limitation of this study lies in the data collection procedure. Methodologically, it was carried out through an online questionnaire, which introduces biases related to the possibility of generalizing the results, since this method meant we only worked with those participants who had access to the Internet. The data analysis was carried out only from a quantitative approach, which reduces the possibility of understanding the phenomenon from the experiences of the participants themselves.
